# FedHGPrompt: Privacy-Preserving Federated Prompt Learning for Few-Shot Heterogeneous Graph Learning

**DOI:** 10.3390/e28020143

**Published:** 2026-01-27

**Authors:** Xijun Wu, Jianjun Shi, Xinming Zhang

**Affiliations:** School of Computer Science and Technology, University of Science and Technology of China, Hefei 230027, China; wuxijun@mail.ustc.edu.cn (X.W.); shijj0817@mail.ustc.edu.cn (J.S.)

**Keywords:** federated learning, heterogeneous graphs, prompt learning, few-shot learning, privacy preservation, secure aggregation

## Abstract

Learning from heterogeneous graphs under the constraints of both data scarcity and data privacy presents a significant challenge. While graph prompt learning offers a pathway for efficient few-shot adaptation, and federated learning provides a paradigm for decentralized training, their direct integration for heterogeneous graphs is non-trivial due to structural complexity and the need for rigorous privacy guarantees. This paper proposes FedHGPrompt, a novel federated framework that bridges this gap through a cohesive architectural design. Our approach introduces a three-layer model: a unification layer employing dual templates to standardize heterogeneous graphs and tasks, an adaptation layer utilizing trainable dual prompts to steer a frozen pre-trained model for few-shot learning, and a privacy layer integrating a cryptographic secure aggregation protocol. This design ensures that the central server only accesses aggregated updates, thereby cryptographically safeguarding individual client data. Extensive evaluations on three real-world heterogeneous graph datasets (ACM, DBLP, and Freebase) demonstrate that FedHGPrompt achieves superior few-shot learning performance compared to existing federated graph learning baselines (including FedGCN, FedGAT, FedHAN, and FedGPL) while maintaining strong privacy assurances and practical communication efficiency. The framework establishes an effective approach for collaborative learning on distributed, heterogeneous graph data where privacy is paramount.

## 1. Introduction

Graph-structured data is ubiquitous in modern applications, ranging from social networks and knowledge graphs to molecular structures and financial transaction networks. A significant portion of these real-world graphs are heterogeneous, involving multiple types of nodes and edges, which encode rich semantic relationships. Learning meaningful representations from such graphs is crucial for downstream tasks like node classification and link prediction. However, two formidable challenges often arise in practice: data scarcity and data privacy.

On the one hand, obtaining sufficient labeled data for training powerful models like graph neural networks (GNNs) is expensive and time-consuming, especially for emerging or long-tail categories. This has spurred significant interest in few-shot learning on graphs. On the other hand, graph data is frequently distributed across multiple parties (e.g., different hospitals, financial institutions, or social platforms) and contains sensitive information. Regulations and competitive concerns prohibit the centralized collection of this data, making federated learning (FL)—which trains models collaboratively without sharing raw data—an appealing paradigm [1]. The intersection of these challenges defines a critical yet underexplored problem: how to perform effective few-shot learning on distributed, private, and heterogeneous graphs?

Recent advances in related fields offer partial solutions. Heterogeneous graph neural networks (HGNNs) [2,3] and their recent extensions that integrate Large Language Models for generalized processing [4] can model complex graph structures. However, they typically require substantial labeled data for end-to-end training. To address data scarcity, prompt learning on graphs has emerged as a parameter-efficient alternative to full fine-tuning [5]. Inspired by successes in natural language processing, it adapts a pre-trained model to downstream tasks by learning lightweight “prompts.” Notable works like GraphPrompt [6] unify pre-training and downstream tasks, and HGPrompt [7] further introduces a dual-prompt mechanism specifically designed for heterogeneous graphs, achieving state-of-the-art few-shot performance.

Concurrently, federated graph learning (FGL) has developed to handle decentralized graph data. Early efforts focused on basic frameworks for graph data [8], with subsequent research addressing non-IID challenges such as latent link-type heterogeneity [9] and personalization [10]. Recent trends include leveraging graph structures for fine-grained client collaboration [11] and employing Large Language Models (LLMs) to mitigate data heterogeneity at the data level [12]. A concurrent work, FedGPL [13], also explores prompt learning within federated graphs to handle heterogeneity. However, these FGL methods either do not leverage the parameter efficiency and rapid adaptability of prompt learning, assume relatively sufficient local data, or, crucially, lack rigorous cryptographic privacy guarantees against a curious central server, often relying on the naive assumption of a “trusted” aggregator.

Therefore, a significant gap persists: There is no framework that simultaneously addresses (1) the structural complexity of heterogeneous graphs, (2) the label scarcity in few-shot learning, and (3) the stringent privacy requirements of federated learning with provable security guarantees.

To bridge this gap, we propose FedHGPrompt, a novel federated heterogeneous graph prompt learning framework. Our core insight is to integrate the representational power of pre-trained HGNNs, the adaptation efficiency of prompt learning, and the privacy protection of secure aggregation within a cohesive, three-layer architecture. First, we leverage a dual-template design (graph template and task template) to unify heterogeneous graphs and reformulate diverse tasks. Second, we equip clients with lightweight dual-prompt vectors (feature and heterogeneity prompts) to adapt a frozen, pre-trained backbone to local few-shot tasks efficiently. Most importantly, to protect client privacy against a curious server, we seamlessly integrate a secure aggregation protocol [14] that cryptographically ensures the server only learns the aggregated prompt updates, not individual contributions.

It is important to note that the core learning mechanism for adapting to heterogeneous graphs and few-shot tasks is inherited from our team’s prior work, the HGPrompt [7] framework. The primary novelty of the present work lies in the system-level integration of this mechanism into a federated learning paradigm with rigorous privacy guarantees, addressing the unique challenges of data decentralization, scarcity, and security simultaneously.

The main contributions of this work are summarized as follows:

- We formally define the problem of federated few-shot learning on heterogeneous graphs and establish a comprehensive threat model with clear privacy objectives.
- We propose FedHGPrompt, the first framework that seamlessly integrates heterogeneous graph prompt learning with a secure aggregation protocol, enabling privacy-preserving and communication-efficient collaborative learning.
- We provide a detailed privacy analysis, demonstrating that our framework protects individual client updates under standard cryptographic assumptions, even in the presence of client dropouts and collusion.
- Through extensive experiments on three public heterogeneous graph datasets (ACM, DBLP, and Freebase), we validate that FedHGPrompt significantly outperforms existing federated graph learning baselines—including both standard federated GNNs and a concurrent federated graph prompt method—in few-shot scenarios while maintaining robust privacy.

The remainder of this paper is organized as follows: Section 2 reviews related work. Section 3 presents preliminaries and formally states our problem. Section 4 details the FedHGPrompt methodology. Section 5 presents experimental results and analysis. Finally, Section 6 concludes the paper and discusses future directions.

## 2. Related Work

This section reviews the research landscape relevant to our proposed FedHGPrompt framework, which intersects three major fields: graph representation learning, the emerging paradigm of prompt learning on graphs, and federated learning over graph-structured data. We discuss the key advancements and identify the existing gaps that motivate our work.

### 2.1. Graph Representation Learning: From Homogeneous to Heterogeneous Graphs

Graph neural networks (GNNs) have become the de facto standard for representation learning on graph-structured data. Early works like graph convolutional networks (GCNs) [15] and Graph Attention Networks (GAT) [16] laid the foundation for learning node embeddings through neighborhood aggregation. However, these models are primarily designed for homogeneous graphs, where a single type of node and edge exists.

Real-world graphs often involve multiple node and edge types, known as heterogeneous graphs. To model such complexity, heterogeneous graph neural networks (HGNNs) have been developed. Models like HAN [2] leverage meta-paths to aggregate semantics, while Simple-HGN [3] extends GAT by incorporating edge-type information during neighborhood aggregation. These models show superior performance on heterogeneous graphs but require extensive task-specific labels for end-to-end training, which are expensive to obtain.

To reduce labeling dependency, self-supervised pre-training on graphs has gained traction. For homogeneous graphs, methods like DGI [17] and GraphCL [18] employ contrastive learning on graph augmentations. For heterogeneous graphs, pre-training techniques such as CPT-HG [19] and HeCo [20] design pretext tasks to capture semantic and structural properties. However, a significant performance gap often exists between the objectives of general pre-training and specific downstream tasks [7]. Recent trends include integrating Large Language Models (LLMs) to enhance semantic understanding in graphs with arbitrary formats [4] and developing robust representation learning methods for noisy heterogeneous graphs [21]. Another advanced direction explores learning in non-Euclidean (e.g., hyperbolic) spaces to better preserve hierarchical graph structures [22]. However, a significant performance gap often exists between the objectives of general pre-training and specific downstream tasks [7]. Our work builds upon these foundations but addresses the critical challenge of adapting pre-trained knowledge to few-shot tasks in a federated and privacy-preserving manner.

### 2.2. Prompt Learning on Graphs

Inspired by the success of prompting in large language models, prompt learning has emerged as a parameter-efficient alternative to full fine-tuning for adapting pre-trained models to downstream tasks. The core idea is to design a task-specific “prompt” that reformulates the downstream task to align with the pre-trained model’s objective, thereby eliciting its relevant knowledge.

Early explorations in graph prompt learning primarily focused on homogeneous graphs. GPPT [23] aligns node classification with a link prediction pre-training task via a prompting module. GraphPrompt [5] proposes a more unified framework by employing a subgraph similarity prediction task as a common template for both pre-training and downstream tasks, using a learnable prompt to bridge the gap. This line of work has been extended by surveys that systematize the field [6,24] and frameworks like MultiGPrompt that exploit multi-task pre-training [25].

A key focus of recent research is prompt learning under data scarcity. A significant challenge is the potential misalignment between the objective of common link-prediction-based pre-training (bringing connected nodes closer) and that of downstream classification (bringing nodes of the same class closer), which can introduce noise and cause negative transfer in few-shot settings [26]. To address this, recent work like Di-Graph [27] proposes an information-bottleneck-driven prompt framework. It employs a diffusion sampling strategy to select more suitable pre-training samples for learning robust representations and designs an information bottleneck objective to filter out irrelevant features captured during pre-training, thereby achieving superior performance in genuine few-shot scenarios. Furthermore, for text-attributed graphs, leveraging LLMs in conjunction with GNNs for pre-training and prompt design has been shown to significantly enhance few-shot node classification by fully utilizing both textual and structural information [28].

A significant advancement is HGPrompt [7], which directly addresses the heterogeneity challenge we focus on. As detailed in Section 3, HGPrompt introduces a dual-template design: a graph template to unify heterogeneous and homogeneous graph formats, and a task template based on subgraph similarity. It further employs a dual-prompt mechanism to bridge gaps caused by feature variations and heterogeneity differences across tasks. Our framework, FedHGPrompt, directly incorporates and extends the HGPrompt architecture as its core learning component. The critical distinction and contribution of our work lie in re-engineering this powerful learning mechanism to operate effectively within the stringent constraints of a federated learning environment where data privacy is paramount and local datasets are non-IID and few-shot.

### 2.3. Federated Learning on Graphs

Federated learning (FL) aims to train a global model collaboratively across decentralized clients without sharing raw data. Federated graph learning (FGL) is a specialized domain within this broader and rapidly evolving field [29,30]. While FL has been extensively studied for Euclidean data, FGL presents unique challenges due to the non-IID nature of graph data, structural dependencies, and privacy risks associated with graph topology. These challenges have spurred the development of dedicated FGL methods, which we categorize and discuss below, with a focus on how they address (or fail to address) heterogeneity, data scarcity, and privacy—the core gaps that FedHGPrompt aims to bridge.

Several works address non-IID challenges in FGL. FedStar [31] shares common structural knowledge across graphs. FGGP [11] tackles domain shift via generalizable prototypes. Other approaches focus on subgraph FL settings [8], employ reinforcement learning for efficient sampling [32], or leverage federated graph-based sampling to handle arbitrary client availability [33]. In federated settings with scarce data, the intersection of personalized federated learning (PFL) and few-shot learning becomes crucial. A common challenge is the high task heterogeneity across decentralized graph data, coupled with extremely limited local training data per client. Traditional PFL methods may produce erroneous predictions due to a uniform classification head, while methods that only locally fine-tune a client-specific classifier are constrained by the scarce data. To tackle this, recent frameworks like FedPANO [10] have been proposed. FedPANO decouples the local model into a shared GNN (federatedly trained to capture cross-client knowledge) and a personalized classifier (locally customized), combined with a node generator policy to mitigate client-side node scarcity, thereby achieving personalized federated few-shot node classification. Another work, FCGNN [34], addresses few-shot graph classification by training a dual-branch network per client to collaboratively represent graphs from different perspectives and incorporates a relational aggregation layer to leverage relational information among graph samples, reducing overfitting and yielding better generalization. A related but distinct line of work has begun to explore the integration of prompting within FL to handle heterogeneity. FedGPL [13], proposed concurrently with our work, is a federated graph prompt learning framework designed to address multifaceted graph and task heterogeneity through asymmetric knowledge transfer and a virtual prompt graph.

However, none of these federated approaches simultaneously satisfy the three core requirements of our target scenario: (1) effective handling of full-scale, heterogeneous graph data, (2) adaptation to few-shot learning tasks on each client, and (3) providing strong, cryptographic privacy guarantees for client updates. The integration of HGPrompt’s sophisticated dual-prompt mechanism with a robust secure aggregation protocol remains unexplored.

### 2.4. Secure Aggregation in Federated Learning

Secure aggregation protocols are essential cryptographic primitives for preventing privacy leakage from individual model updates in FL. The foundational protocol by Bonawitz et al. [14] enables a server to compute the sum of high-dimensional user updates while guaranteeing that nothing beyond the aggregated sum is learned, even in the presence of client dropouts. It achieves this through a combination of pairwise masking with Diffie–Hellman keys, Shamir’s secret sharing, and a double-masking technique for dropout resilience. This work established the standard for synchronous, single-server secure aggregation. Subsequent research has extended this paradigm along several critical dimensions: improving communication efficiency and robustness against stronger adversaries, integrating with other privacy techniques like differential privacy (DP), and adapting to novel FL architectures.

A significant line of work focuses on enhancing the practicality and robustness of the core cryptographic protocol. For instance, SeaFlame [35] addresses the threat of malicious participants by employing two non-colluding servers and optimized share conversion techniques to improve communication efficiency. Similarly, to tackle the substantial communication overhead in constrained environments like the Internet of Things (IoT), protocols such as Hyb-Agg [36] have been proposed. Hyb-Agg combines homomorphic encryption with masking to reduce the aggregation process to a single, non-interactive client transmission, ensuring constant per-client communication. Other approaches, like TAPFed [37], utilize threshold functional encryption to secure the aggregation process against inference attacks from curious or malicious aggregators in decentralized settings.

Another crucial dimension is the integration of secure aggregation with differential privacy (DP). While secure aggregation protects updates from the server, DP provides formal, information-theoretic guarantees against inference attacks based on the final model or aggregated outputs. Recent works strive to balance the trade-off between privacy and model utility inherent in DP. For example, DP-FedASGP [38] mitigates excessive noise by identifying and protecting significant gradients during aggregation, thereby improving model accuracy under strict privacy budgets. Furthermore, algorithms like DP-FedCMRS [39] combine DP with client clustering to address the dual challenge of data heterogeneity and privacy, showcasing how these techniques can be jointly optimized.

The landscape of FL itself is evolving, moving beyond simple synchronous settings. This necessitates the development of new secure aggregation protocols. Buffalo [40] represents a key advancement as a practical protocol designed for Buffered Asynchronous FL (BAsyncFL), utilizing lattice-based encryption to handle stragglers without compromising security. Similarly, solutions like RaSA [41] are designed for hierarchical FL architectures, offering robust and adaptive security for edge-assisted environments. These works highlight the ongoing adaptation of secure aggregation to increasingly complex and realistic FL paradigms.

While the protocol by Bonawitz et al. [14] is generic and model-agnostic, its direct application to a sophisticated, prompt-based graph learning framework like HGPrompt is non-trivial. The dual-prompt structure (feature and heterogeneity prompts) and the associated graph template operations introduce specific communication patterns and vector formats. These must be seamlessly integrated into the multi-round secure aggregation protocol without breaking the security guarantees or incurring prohibitive overhead. Furthermore, the few-shot, heterogeneous, and non-IID nature of the local graph data in our target scenario amplifies the need for an aggregation scheme that is both privacy-preserving and communication-efficient. Our work, FedHGPrompt, provides this crucial integration. We leverage the robustness and dropout resilience of the Bonawitz et al. protocol as our foundation, while carefully designing the interaction between the client’s local HGPrompt training and the secure aggregation rounds. This creates a cohesive system where the advanced representational and adaptive capabilities of HGPrompt are preserved within a privacy-by-design federated protocol.

### 2.5. Summary and Positioning

Table 1 summarizes the landscape and positions our contribution. While significant progress has been made independently in graph prompt learning (especially for heterogeneity) and federated learning, their intersection remains sparse. Existing federated graph learning methods either do not employ prompting, do not fully address graph heterogeneity, or lack strong privacy guarantees against a curious server. Our proposed FedHGPrompt is the first framework, to our knowledge, that integrates the heterogeneous graph prompting power of HGPrompt with the rigorous privacy protection of secure aggregation, specifically tailored for the challenging few-shot federated learning scenario. This integration addresses a critical gap in enabling collaborative, knowledge-rich, and privacy-preserving learning on distributed heterogeneous graph data.

## 3. Preliminaries and Problem Statement

### 3.1. Graph Representation and Few-Shot Learning Background

#### 3.1.1. Heterogeneous Graph Formalization

We consider heterogeneous information networks as the fundamental data structure throughout this work. Formally, a heterogeneous graph is defined as $H=(V,E,T_{V},T_{E},\phi ,\psi )$, where V represents the vertex set and E denotes the edge set. $T_{V}$ and $T_{E}$ are finite sets of node and edge types, respectively, with mapping functions $\phi :V\to T_{V}$ and $\psi :E\to T_{E}$ assigning types to vertices and edges. A homogeneous graph constitutes a special case where $|T_{V}\left|=\right|T_{E}|=1$, indicating uniform node and edge types. Each vertex v∈V is associated with a feature vector $x_{v}\in R^{d_{\mathrm{feat}}}$ capturing its intrinsic attributes.

#### 3.1.2. Few-Shot Learning on Graphs

In the few-shot learning paradigm considered in this paper, we focus on *K*-shot learning scenarios where each target class has exactly *K* labeled examples available for model adaptation. A learning task T is defined as T=(S,Q), where $S={\left\{\left(x_{i},y_{i}\right)\right\}}_{i=1}^{|C|\times K}$ denotes the support set containing *K* labeled examples per class from the class set C, and $Q={\left\{x_{j}\right\}}_{j=1}^{M}$ represents the query set comprising unlabeled instances requiring prediction. The objective is to leverage the limited supervision in S to accurately predict labels for instances in Q, necessitating effective knowledge transfer and rapid adaptation capabilities.

#### 3.1.3. Graph Neural Networks and Prompt Learning

Graph neural networks (GNNs) have emerged as the predominant architecture for learning representations on graph-structured data. A typical GNN layer employs a message-passing mechanism where each node aggregates information from its neighbors, followed by a non-linear transformation. However, conventional GNNs trained in a fully supervised manner require substantial labeled data, which is often unavailable in real-world scenarios. To address this limitation, prompt learning has been recently introduced as a parameter-efficient adaptation strategy. In this paradigm, a pre-trained GNN backbone remains frozen, while lightweight prompt vectors are optimized to align downstream tasks with the knowledge encapsulated in the pre-trained model, significantly reducing the need for extensive labeled data.

### 3.2. Federated Learning System Architecture

#### 3.2.1. System Participants and Data Distribution

We consider a federated learning ecosystem comprising a central coordination server S and a collection of *N* distributed clients $\left\{C_{1},C_{2},\ldots ,C_{N}\right\}$. Each client $C_{i}$ possesses a private heterogeneous graph $H_{i}=\left(V_{i},E_{i},T_{V,i},T_{E,i},\phi_{i},\psi_{i}\right)$ that represents its local data. Importantly, these graphs are non-independent and identically distributed (non-IID) across clients, reflecting diverse real-world scenarios where different organizations or users maintain data with varying distributions, graph topologies, and node/edge type compositions. Associated with each client is a few-shot learning task $T_{i}=\left(S_{i},Q_{i}\right)$ defined on its private graph, where the support set $S_{i}$ contains *K* labeled examples per class and the query set $Q_{i}$ consists of unlabeled instances.

#### 3.2.2. Federated Training Process

The federated learning process operates through synchronized communication rounds indexed by t=1,2,…,T. In each round *t*, a subset of clients $P^{\left(t\right)}\subseteq \left\{C_{1},\ldots ,C_{N}\right\}$ is selected to participate in training, typically based on availability or sampling strategies. The global model, maintained by the server, is characterized by parameters Ω=[Θ,Π], which comprise two distinct components: (1) Θ denotes the parameters of a pre-trained graph neural network backbone that remains frozen throughout federated training, and (2) Π=[P,Q] represents trainable prompt vectors, where $P\in R^{d_{\mathrm{prompt}}}$ serves as the feature prompt and $Q\in R^{|T_{V}|+1}$ functions as the heterogeneity prompt.

#### 3.2.3. Global Learning Objective

The overarching goal of federated training is to learn a global set of prompt vectors Π that minimize the aggregate loss across all clients, while keeping the pre-trained backbone Θ fixed. Formally, the optimization problem is defined as

(1) $$\min_{\Pi }F\left(\Pi \right)=\sum_{i=1}^{N}\frac{|D_{i}|}{\sum_{j=1}^{N}\left|D_{j}\right|}L_{i}\left(\Theta ,\Pi ;D_{i}\right),$$

where $D_{i}$ represents the private dataset of client $C_{i}$, typically derived from its support set $S_{i}$ or query set $Q_{i}$, and $L_{i}$ denotes the local loss function specific to client $C_{i}$. The weighting coefficient $|D_{i}|/\sum_{j}\left|D_{j}\right|$ ensures that clients with larger datasets contribute proportionally more to the global objective.

#### 3.2.4. Communication and Aggregation Mechanism

During each communication round *t*, the server broadcasts the current global prompt vectors $\Pi^{\left(t\right)}$ to all participating clients in $P^{\left(t\right)}$. Each client $C_{i}\in P^{\left(t\right)}$ then performs local training on its private data to compute updated prompt vectors $\Pi_{i}^{\left(t+1\right)}$. Instead of transmitting the raw updates $\Delta \Pi_{i}^{\left(t\right)}=\Pi_{i}^{\left(t+1\right)}-\Pi^{\left(t\right)}$ directly to the server—which would compromise data privacy—clients engage in a secure aggregation protocol. This protocol ensures that the server only learns the aggregated update ${\bar{\Delta \Pi }}^{\left(t\right)}=\sum_{i\in P^{\left(t\right)}}w_{i}\Delta \Pi_{i}^{\left(t\right)}$, where $w_{i}$ are client-specific weights typically proportional to dataset sizes. The server subsequently updates the global prompts as $\Pi^{\left(t+1\right)}=\Pi^{\left(t\right)}+\eta {\bar{\Delta \Pi }}^{\left(t\right)}$, with η denoting the global learning rate.

### 3.3. Threat Model and Security Objectives

#### 3.3.1. Adversarial Capabilities and Assumptions

We consider the following two primary categories of adversaries within our federated learning framework, each with distinct capabilities and objectives:

1. Honest-but-Curious (Passive) Adversaries:
  - All protocol participants, including the server and clients, adhere strictly to the prescribed protocol steps without deviation.
  - However, adversaries may attempt to infer sensitive information about other participants’ private data by analyzing all messages exchanged during protocol execution.
  - We allow for collusion among up to f<N clients and the server, where these entities may pool their views to enhance inference capabilities.
  - The pre-trained model Θ and global prompts Π are considered public knowledge, as they are distributed to all clients.
2. Active (Malicious) Adversaries:
  - Adversaries may arbitrarily deviate from the protocol specification to achieve their objectives.
  - The server may drop, modify, delay, or inject fabricated messages to disrupt training or extract private information.
  - Malicious clients may submit malformed or manipulated updates to compromise model integrity or launch poisoning attacks.
  - Clients may drop out unexpectedly during protocol execution due to network failures or adversarial behavior.
  - We assume the existence of a Public Key Infrastructure (PKI) that enables authentication of messages, preventing the server from impersonating legitimate clients.

#### 3.3.2. Privacy and Security Goals

Our framework aims to achieve the following security properties:

Input Privacy: For any coalition of up to *f* clients colluding with the server, the joint view of the protocol execution should reveal no information about the private data of honest clients beyond what can be inferred from the aggregated model updates. Formally, there should exist a probabilistic polynomial-time simulator Sim such that for all admissible adversary ensembles A, the following computational indistinguishability holds:

(2) $${\mathrm{View}}_{A}^{\mathrm{Real}}\left({\left\{H_{i},T_{i}\right\}}_{i=1}^{N}\right)\approx_{c}{\mathrm{Sim}}_{A}\left({\left\{{\bar{\Delta \Pi }}^{\left(t\right)}\right\}}_{t=1}^{T}\right),$$

where ${\mathrm{View}}_{A}^{\mathrm{Real}}$ represents the adversary’s view in a real protocol execution, and $\left\{{\bar{\Delta \Pi }}^{\left(t\right)}\right\}$ denotes the sequence of aggregated updates.

Robustness to Dropouts: The protocol should maintain correctness and privacy even when a subset of clients fail to complete the protocol due to network failures or adversarial dropout. Specifically, as long as at least *t* clients (where *t* is a security threshold) complete the protocol, the server should be able to compute the correct aggregated update without compromising the privacy of dropped clients’ data.

Integrity Against Active Attacks: In the active adversary model, the protocol should ensure that malicious participants cannot undetectably alter the computation results beyond contributing arbitrary inputs. While we do not focus on detecting or mitigating Byzantine failures in this work, we ensure that active adversaries cannot learn additional private information beyond what passive adversaries could obtain.

#### 3.3.3. Differential Privacy Considerations

Although not the primary focus of this work, our framework can be seamlessly integrated with differential privacy mechanisms to provide stronger privacy guarantees. By adding carefully calibrated noise to local updates before secure aggregation, we can achieve (ϵ,δ)-differential privacy, ensuring that the participation of any individual client does not significantly affect the aggregated result. This composition provides formal privacy guarantees against strong adversaries with arbitrary background knowledge.

## 4. Methodology

### 4.1. System Architecture Overview

FedHGPrompt is a novel framework designed for federated few-shot learning on heterogeneous graphs. It is built upon our prior work, HGPrompt [7], for heterogeneous graph prompt learning. We inherit its dual-template design for graph unification and task formulation, as well as its dual-prompt mechanism for feature and heterogeneity adaptation. The key extension and contribution of this work is the re-engineering of this learning paradigm into a privacy-preserving federated system. Specifically, we design the federated training workflow around the lightweight prompts and integrate a cryptographic secure aggregation protocol to protect client updates, thereby addressing the critical challenges of data privacy and decentralization that are not considered in the original HGPrompt.

**Figure 1:**
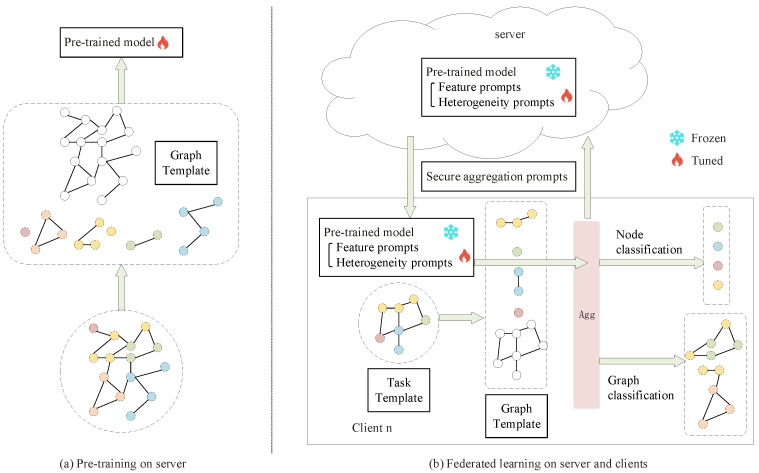
The FedHGPrompt framework. (**a**) Pre-training phase: A GNN model is pre-trained on the server to capture general graph knowledge. (**b**) Federated learning phase: The pre-trained model is frozen and distributed. Lightweight prompt vectors are initialized and then collaboratively learned across clients via secure aggregation.

As illustrated in Figure 1, the framework operates in two distinct phases:

(a) Pre-training phase: A graph neural network (GNN) model is first pre-trained on the server using available graph data. This phase produces a powerful and general-purpose frozen GNN backbone model Θ, which serves as a shared feature extractor for all subsequent federated tasks. No prompt vectors are involved at this stage.
(b) Federated learning phase: This is the core adaptive learning stage. The pre-trained backbone Θ is distributed to all clients and remains fixed. The federated learning process focuses on optimizing a set of lightweight, learnable prompt vectors Π=[P,Q], which are initialized at the start of this phase. The process unfolds as follows:
  - Local Adaptation with Dual Templates: On each client, the private heterogeneous graph is unified via a Graph Template, and the few-shot task is formulated via a Task Template.
  - Prompt Tuning: Clients adapt the global prompts locally by tuning them on their private few-shot support sets. Only the prompts ($P_{i},Q_{i}$) are updated, keeping the backbone Θ frozen.
  - Secure Aggregation: The server collects and securely aggregates the local prompt updates to obtain a refined global prompt set Π, ensuring no leakage of individual client data.

This decoupled design enables efficient and privacy-preserving knowledge transfer: the frozen pre-trained backbone provides rich, general graph representations, while the federated prompts learn to adapt these representations to diverse downstream tasks across clients.

Discussion on the Pre-trained Backbone Assumption. The two-phase design relies on a centrally pre-trained backbone model Θ. A critical clarification is that this pre-training, like common practice in CV, NLP, and graph self-supervised learning, utilizes only unlabeled graph data (e.g., for link prediction). Obtaining such unlabeled structural data is often more feasible and raises significantly fewer privacy concerns than collecting labeled task-specific data. Therefore, FedHGPrompt is well-suited for cross-silo scenarios where (1) participants can share non-sensitive, unlabeled graph data (e.g., anonymized user interaction logs, public citation networks) to jointly pre-train a powerful foundation model; or (2) a relevant pre-trained model from public domains (like academic graph benchmarks) is available as a starting point. The federated phase then operates on the private, labeled few-shot data where privacy is paramount. We acknowledge that in the strictest federated settings where any data centralization is prohibited, this assumption poses a challenge. For such cases, the architecture of FedHGPrompt naturally extends to future work exploring federated self-supervised pre-training of the backbone across clients, after which the prompt learning phase would proceed as described.

### 4.2. Graph Unification via Dual Templates

#### 4.2.1. Graph Structure Template

To address the challenge of heterogeneous graph structures in federated learning, we propose a graph template mechanism that transforms heterogeneous graphs into a collection of homogeneous views. Consider a heterogeneous graph $H=(V,E,T_{V},T_{E},\phi ,\psi )$ where V is the vertex set, E is the edge set, $T_{V}$ and $T_{E}$ are sets of node and edge types, respectively, and $\phi :V\to T_{V}$, $\psi :E\to T_{E}$ are type mapping functions.

The graph template transformation $\Gamma :H\to \{G_{0},G_{1},\ldots ,G_{|T_{V}|}\}$ produces $|T_{V}|+1$ homogeneous graphs,

(3) $$\Gamma \left(H\right)=\left\{G_{0}\right\}\cup \left\{G_{t}|t\in T_{V}\right\},$$

where the following apply:

- $G_{0}=\left(V,E\right)$ preserves the complete graph topology without type distinctions, capturing the overall connectivity patterns.
- For each node type $t\in T_{V}$, $G_{t}=\left(V_{t},E_{t}\right)$ with $V_{t}=\left\{v\in V|\phi \left(v\right)=t\right\}$ and $E_{t}=\left\{\left(u,v\right)\in E|\phi \left(u\right)=\phi \left(v\right)=t\right\}$, representing the subgraph induced by nodes of type *t* and edges between them.

This transformation serves two critical purposes. First, it enables the application of homogeneous graph neural networks (which require uniform node and edge types) to heterogeneous graphs. Second, by preserving type-specific subgraphs separately, it maintains the ability to distinguish between different semantic aspects of the original heterogeneous graph.

#### 4.2.2. Task Formulation Template

To unify different downstream tasks across clients, we employ a task template based on subgraph similarity prediction. For any graph element (node, edge, or entire graph) *x*, we first extract its context subgraph C(x) as follows:

- For node-level tasks, C(v) is the δ-hop neighborhood subgraph centered at node *v*.
- For graph-level tasks, C(G)=G (the entire graph).

The embedding of this context subgraph is computed through a multi-view aggregation process,

(4) $$z_{x}=\mathrm{Aggregate}\left(\left\{\mathrm{Readout}\left(G_{t}\left[C\left(x\right)\right]\right)|G_{t}\in \Gamma \left(C\left(x\right)\right)\right\}\right),$$

where $G_{t}\left[C\left(x\right)\right]$ denotes the projection of the context subgraph onto the homogeneous graph view $G_{t}$, Readout is a function that aggregates node embeddings within a subgraph into a single vector, and Aggregate combines embeddings from different type-specific views.

The following three fundamental graph learning tasks are reformulated within this unified framework:

1. Link Prediction: Given a positive edge $\left(u,v^{+}\right)$ and a negative candidate $\left(u,v^{-}\right)$, the objective becomes
  (5) $$\mathrm{sim}\left(z_{u},z_{v^{+}}\right)>\mathrm{sim}\left(z_{u},z_{v^{-}}\right),$$
  where sim(·,·) is a similarity function (e.g., cosine similarity).
2. Node Classification: For a node *v* with class label *c*, we construct class prototypes $\mu_{c}$ as the average embedding of support nodes from class *c*,
  (6) $$\mu_{c}=\frac{1}{K}\sum_{(x,y)\in S,y=c}z_{x},$$
  where S is the support set and *K* is the number of shots. The classification rule is
  (7) $$\hat{c}=\arg\max_{c\in C}\mathrm{sim}\left(z_{v},\mu_{c}\right).$$
3. Graph Classification: Similarly to node classification, but operating on graph-level embeddings $z_{G}$ and graph class prototypes.

This unified formulation enables consistent training objectives across different tasks and facilitates knowledge transfer in the federated setting.

### 4.3. Federated Prompt Learning Formulation

#### 4.3.1. Problem Formalization

We consider a federated learning system with one central server S and *N* clients $\left\{C_{1},C_{2},\ldots ,C_{N}\right\}$. Each client $C_{i}$ possesses a private heterogeneous graph $H_{i}$ and a few-shot learning task $T_{i}=\left(S_{i},Q_{i}\right)$, where $S_{i}$ is the support set with *K* labeled examples per class, and $Q_{i}$ is the query set.

The learning objective is to train a global model parameterized by Ω=[Θ,Π], where Θ represents the parameters of a pre-trained graph neural network backbone (kept frozen during federated training), and Π=[P,Q] represents trainable prompt vectors with P as feature prompts and Q as heterogeneity prompts.

The global optimization problem is

(8) $$\min_{\Pi }F\left(\Pi \right)=\sum_{i=1}^{N}\frac{|D_{i}|}{\sum_{j=1}^{N}\left|D_{j}\right|}L_{i}\left(\Theta ,\Pi ;D_{i}\right),$$

where $D_{i}$ is client *i*’s private dataset, and $L_{i}$ is the local loss function defined as

(9) $$L_{i}\left(\Theta ,\Pi ;D_{i}\right)=-\frac{1}{|D_{i}|}\sum_{\left(x,y\right)\in D_{i}}\log\frac{\exp(\mathrm{sim}\left(z_{x},\mu_{y}\right)/\tau )}{\sum_{c\in C}\exp\left(\mathrm{sim}\left(z_{x},\mu_{c}\right)/\tau \right)},$$

with τ as a temperature parameter and $\mu_{c}$ as the class prototype for class *c*.

#### 4.3.2. Dual-Prompt Design: Feature and Heterogeneity Prompts

To effectively bridge the gap between pre-training tasks and downstream tasks, we propose a dual-prompt mechanism comprising feature prompts and heterogeneity prompts. These prompts enable task-specific adaptation of the frozen pre-trained model by modulating feature importance and handling semantic variations arising from graph heterogeneity.

Feature Prompt for Readout. The feature prompt $P\in R^{d_{\mathrm{prompt}}}$ is applied before the readout operation to modulate node embeddings, allowing different downstream tasks to focus on different aspects of the learned representations. Let $h_{v}\in R^{d}$ denote the embedding of node *v* obtained from the pre-trained GNN backbone. The feature prompt performs element-wise multiplication on $h_{v}$,

$${\tilde{h}}_{v}=P⊙h_{v},$$

where ⊙ denotes element-wise multiplication. This modulated embedding is then used as input to the readout function. By adjusting the importance of different dimensions in the node embeddings, the feature prompt enables task-specific feature selection without retraining the backbone model.

Heterogeneity Prompt. The heterogeneity prompt $Q\in R^{|T_{V}|+1}$ is designed to weight the contributions of different homogeneous subgraphs derived from the graph template. After applying the graph template, we obtain $|T_{V}|+1$ homogeneous subgraphs $G_{0},G_{1},\ldots ,G_{|T_{V}|}$. For each subgraph, we compute a graph-level representation via a readout operation over the feature-prompt-modulated node embeddings. The final representation is a weighted sum,

$$z=\sum_{t=0}^{|T_{V}|}Q\left[t\right]\cdot \mathrm{Readout}\left(\left\{{\tilde{h}}_{v}|v\in V\left(G_{t}\right)\right\}\right),$$

where Q[t] is the learnable weight for subgraph $G_{t}$. This enables the model to adaptively fuse information from different node-type-specific views according to task needs.

Readout Operation with Feature Prompt. The readout operation aggregates modulated node embeddings within a subgraph into a single graph-level vector. Given a homogeneous subgraph $G_{t}$ with node embeddings ${\left\{{\tilde{h}}_{v}\right\}}_{v\in V(G_{t})}$ (already modulated by the feature prompt), we define the readout as

$$\mathrm{Readout}\left(\left\{{\tilde{h}}_{v}|v\in V\left(G_{t}\right)\right\}\right)=\frac{1}{\left|V(G_{t})\right|}\sum_{v\in V(G_{t})}{\tilde{h}}_{v}.$$

This operation is applied independently to each homogeneous subgraph. The feature prompt thus directly influences the readout by reweighting the node embeddings before aggregation.

#### 4.3.3. Federated Aggregation Mechanism

The federated training process operates in synchronized rounds t=1,2,…,T. In each round *t*, the following steps occur:

1. Global Prompt Broadcast: The server distributes the current global prompt vectors $\Pi^{\left(t\right)}=\left[P^{\left(t\right)},Q^{\left(t\right)}\right]$ to all participating clients.
2. Client Local Update: Each client $C_{i}$ performs local training on its private data to compute prompt updates,
  (10) $$\Delta \Pi_{i}^{\left(t\right)}=\Pi_{i}^{\left(t+1\right)}-\Pi^{\left(t\right)},$$
  where $\Pi_{i}^{\left(t+1\right)}$ is obtained by minimizing $L_{i}$ on $D_{i}$ for *E* local epochs.
3. Secure Aggregation: Clients participate in a secure aggregation protocol that computes the weighted sum of updates without revealing individual contributions,
  (11) $${\bar{\Delta \Pi }}^{\left(t\right)}=\sum_{i\in U_{t}}w_{i}\Delta \Pi_{i}^{\left(t\right)},$$
  where $U_{t}\subseteq \left\{1,\ldots ,N\right\}$ is the set of clients participating in round *t*, and $w_{i}=|D_{i}|/\sum_{j\in U_{t}}\left|D_{j}\right|$ are normalized weights based on dataset sizes.
4. Global Prompt Update: The server updates the global prompts,
  (12) $$\Pi^{\left(t+1\right)}=\Pi^{\left(t\right)}+\eta {\bar{\Delta \Pi }}^{\left(t\right)},$$
  where η is a global learning rate.

This formulation ensures that the server only accesses aggregated information, providing privacy guarantees while enabling effective collaborative learning.

### 4.4. Algorithmic Components

#### 4.4.1. Client Local Prompt Training Algorithm

Algorithm 1 details the local training procedure executed by each client. The algorithm takes as input the pre-trained model Θ, current global prompt vectors $P^{\left(t\right)}$ and $Q^{\left(t\right)}$, the client’s private heterogeneous graph $H_{i}$, and the few-shot task specification $T_{i}=\left(S_{i},Q_{i}\right)$.
**Algorithm 1** Client Local Prompt Training**Require:** 
Pre-trained model parameters Θ, global prompts $P^{\left(t\right)},Q^{\left(t\right)}$, private graph $H_{i}$, few-shot task $T_{i}=\left(S_{i},Q_{i}\right)$, local epochs *E*, learning rate $\eta_{l}$**Ensure:** 
Local prompt updates $\Delta P_{i},\Delta Q_{i}$  1:Initialize local prompts: $P_{i}\leftarrow P^{\left(t\right)}$, $Q_{i}\leftarrow Q^{\left(t\right)}$  2:Apply graph template: $\left\{G_{0},\ldots ,G_{m}\right\}\leftarrow \Gamma \left(H_{i}\right)$  3:Extract support set embeddings:  4:**for** each $\left(x,y\right)\in S_{i}$ **do**  5:    Extract context subgraph: $C_{x}\leftarrow \mathrm{ExtractContext}\left(x,H_{i}\right)$  6:    Apply graph template: $\left\{G_{0}\left[C_{x}\right],\ldots ,G_{m}\left[C_{x}\right]\right\}\leftarrow \Gamma \left(C_{x}\right)$  7:    **for** each view j=0 to *m* **do**  8:        Compute view embedding: $e_{x}^{\left(j\right)}\leftarrow {\mathrm{GNN}}_{\Theta }\left(G_{j}\left[C_{x}\right],P_{i}\right)$  9:    **end for**10:    Combine view embeddings: $z_{x}\leftarrow \mathrm{Aggregate}\left(\left\{e_{x}^{\left(0\right)},\ldots ,e_{x}^{\left(m\right)}\right\},Q_{i}\right)$11:    Store embedding: $Z_{x}\leftarrow z_{x}$12:**end for**13:Compute class prototypes:14:**for** each class c∈C **do**15:    $\mu_{c}\leftarrow \frac{1}{K}\sum_{\left(x,y\right)\in S_{i},y=c}Z_{x}$16:**end for**17:**for** epoch =1 to *E* **do**18:    Shuffle query set $Q_{i}$19:    **for** each batch $B\subset Q_{i}$ **do**20:        Initialize gradients: $\nabla_{P_{i}}L\leftarrow 0$, $\nabla_{Q_{i}}L\leftarrow 0$21:        **for** each (x,y)∈B **do**22:           Compute query embedding $z_{x}$ using steps 4–1023:           Compute similarities: $s_{c}\leftarrow \mathrm{sim}\left(z_{x},\mu_{c}\right)$ for all c∈C24:           Compute loss: $\ell \leftarrow -\log\frac{\exp(s_{y}/\tau )}{\sum_{c\in C}\exp\left(s_{c}/\tau \right)}$25:           Accumulate gradients: $\nabla_{P_{i}}L\leftarrow \nabla_{P_{i}}L+\nabla_{P_{i}}\ell$26:           Accumulate gradients: $\nabla_{Q_{i}}L\leftarrow \nabla_{Q_{i}}L+\nabla_{Q_{i}}\ell$27:        **end for**28:        Update prompts: $P_{i}\leftarrow P_{i}-\eta_{l}\nabla_{P_{i}}L$29:        Update prompts: $Q_{i}\leftarrow Q_{i}-\eta_{l}\nabla_{Q_{i}}L$30:    **end for**31:**end for**32:Compute updates: $\Delta P_{i}\leftarrow P_{i}-P^{\left(t\right)}$, $\Delta Q_{i}\leftarrow Q_{i}-Q^{\left(t\right)}$33:**return** $\Delta P_{i},\Delta Q_{i}$

The algorithm begins by initializing local prompt vectors with the global values. It then applies the graph template to transform the private heterogeneous graph into multiple homogeneous views. For each example in the support set, the algorithm extracts the context subgraph, applies the graph template to obtain multiple views, computes embeddings for each view using the pre-trained GNN modulated by the feature prompt $P_{i}$, and finally combines these view embeddings using the heterogeneity prompt $Q_{i}$. Class prototypes are computed as the average embeddings of support examples from each class.

During the local training phase, the algorithm iterates over the query set for *E* epochs. For each batch, it computes embeddings for query examples, calculates similarities to class prototypes, computes the cross-entropy loss, and accumulates gradients with respect to both prompt vectors. The prompts are updated using gradient descent with learning rate $\eta_{l}$. Finally, the algorithm returns the difference between the locally optimized prompts and the initial global prompts as the client’s update.

This local training procedure is designed to be efficient in the few-shot setting, requiring only forward passes through the pre-trained model and gradient computations for the low-dimensional prompt vectors.

#### 4.4.2. Server-Side Prompt Aggregation Algorithm

Algorithm 2 presents the server-side prompt aggregation procedure. In each federated round *t*, the server receives prompt updates $\Delta P_{i}$ and $\Delta Q_{i}$ from each participating client $i\in U_{t}$. The server aggregates these updates using client-specific weights $w_{i}$, which are typically proportional to the client’s dataset size or can be uniform if datasets are balanced. The aggregated updates are then applied to the current global prompts with a global learning rate η, producing the updated global prompts for the next round. This simple yet effective aggregation mechanism enables collaborative learning while maintaining the efficiency of the prompt-based adaptation approach.
**Algorithm 2** Server-Side Prompt Aggregation**Require:** 
Participating clients $U_{t}$, client prompt updates ${\left\{\left(\Delta P_{i},\Delta Q_{i}\right)\right\}}_{i\in U_{t}}$, current global prompts $P^{\left(t\right)},Q^{\left(t\right)}$, aggregation weights ${\left\{w_{i}\right\}}_{i\in U_{t}}$, global learning rate η**Ensure:** 
Updated global prompts $P^{\left(t+1\right)},Q^{\left(t+1\right)}$  1:Initialize aggregated updates: $\bar{\Delta P}\leftarrow 0,\bar{\Delta Q}\leftarrow 0$  2:**for** each client $i\in U_{t}$ **do**  3:    Receive client updates $\Delta P_{i},\Delta Q_{i}$  4:    Weighted aggregation: $\bar{\Delta P}\leftarrow \bar{\Delta P}+w_{i}\cdot \Delta P_{i}$  5:    Weighted aggregation: $\bar{\Delta Q}\leftarrow \bar{\Delta Q}+w_{i}\cdot \Delta Q_{i}$  6:**end for**  7:Update global prompts: $P^{\left(t+1\right)}\leftarrow P^{\left(t\right)}+\eta \cdot \bar{\Delta P}$  8:Update global prompts: $Q^{\left(t+1\right)}\leftarrow Q^{\left(t\right)}+\eta \cdot \bar{\Delta Q}$  9:**return** $P^{\left(t+1\right)},Q^{\left(t+1\right)}$

#### 4.4.3. Secure Aggregation Protocol

Algorithm 3 presents the secure aggregation protocol that protects individual client updates during federated training. The protocol implements a four-round interactive process between clients and the server, incorporating cryptographic primitives to ensure privacy.

The protocol begins with a setup phase where the server establishes cryptographic parameters (lines 1–3). In Round 1 (Key Advertisement, lines 4–10), each client generates encryption and signature key pairs, signs their encryption public key, and sends it to the server. The server verifies all signatures and broadcasts the list of verified clients to establish authenticated identities.

Round 2 (Masked Update Submission, lines 11–30) is the core privacy-preserving step. Each client generates a random self-mask $r_{i}$ and pairwise mask seeds $s_{i,j}$ for every other client using Diffie–Hellman key exchange (lines 13–18). The client then creates Shamir secret shares of these masks, ensuring that at least *t* shares are required for reconstruction (lines 19–22). The client’s actual update $\Delta \Pi_{i}$ is masked using pseudorandom values derived from these masks (line 24), creating ${\tilde{\Delta \Pi }}_{i}$. The masked update and encrypted shares are sent to the server (lines 25–30).
**Algorithm 3** Secure Aggregation Protocol**Require:** 
Client updates ${\left\{\Delta \Pi_{i}\right\}}_{i=1}^{N}$, security threshold *t*, security parameter λ, modulus *M***Ensure:** 
Aggregated update $\bar{\Delta \Pi }$  1:**Setup Phase:**  2:Server generates cryptographic parameters: cyclic group G of prime order *q*, generator *g*, hash function $H:{\left\{0,1\right\}}^{*}\to {\left\{0,1\right\}}^{\lambda }$  3:Server publishes parameters: (G,g,q,H,M,t)  4:**Round 1: Key Advertisement**  5:**for** each client i=1 to *N* **do**  6:    Generate key pairs: $\left(pk_{i}^{enc},sk_{i}^{enc}\right)\leftarrow \mathrm{DH}.\mathrm{KeyGen}\left(G,g\right)$, $\left(pk_{i}^{sig},sk_{i}^{sig}\right)\leftarrow \mathrm{SIG}.\mathrm{KeyGen}\left(\lambda \right)$  7:    Create signature: $\sigma_{i}\leftarrow \mathrm{SIG}.\mathrm{Sign}\left(sk_{i}^{sig},pk_{i}^{enc}\right)$  8:    Send $\left(pk_{i}^{enc},pk_{i}^{sig},\sigma_{i}\right)$ to server  9:**end for**10:Server verifies signatures, compiles list $L={\left\{\left(i,pk_{i}^{enc},pk_{i}^{sig}\right)\right\}}_{i=1}^{N}$, broadcasts L11:**Round 2: Masked Update Submission**12:**for** each client i=1 to *N* **do**13:    Parse L, verify all signatures14:    Generate random seeds: $r_{i}\overset{\$}{\leftarrow }{\left\{0,1\right\}}^{\lambda }$ (self-mask)15:    **for** each other client j≠i **do**16:        Compute shared secret: $k_{i,j}\leftarrow H\left(\mathrm{DH}.\mathrm{SharedSecret}\left(sk_{i}^{enc},pk_{j}^{enc}\right)\right)$17:        Generate pairwise mask seed: $s_{i,j}\leftarrow \mathrm{KDF}\left(k_{i,j},i∥j\right)$18:    **end for**19:    Generate Shamir shares: ${\left\{\left(j,r_{i,j}\right)\right\}}_{j=1}^{N}\leftarrow \mathrm{Shamir}.\mathrm{Share}\left(r_{i},t,N\right)$20:    **for** each j≠i **do**21:        Generate Shamir shares: ${\left\{\left(k,s_{i,j,k}\right)\right\}}_{k=1}^{N}\leftarrow \mathrm{Shamir}.\mathrm{Share}\left(s_{i,j},t,N\right)$22:    **end for**23:    Compute masked update:24:    ${\tilde{\Delta \Pi }}_{i}\leftarrow \Delta \Pi_{i}+\mathrm{PRG}\left(r_{i}\right)+\sum_{j<i}\mathrm{PRG}\left(s_{i,j}\right)-\sum_{j>i}\mathrm{PRG}\left(s_{j,i}\right)\;\mathrm{mod}\;M$25:    **for** each j≠i **do**26:        Encrypt share: $c_{i,j}\leftarrow \mathrm{AE}.\mathrm{Enc}(k_{i,j},i∥j∥r_{i,j}∥{\left\{s_{i,j,k}\right\}}_{k=1}^{N})$27:    **end for**28:    Send $\left({\tilde{\Delta \Pi }}_{i},{\left\{c_{i,j}\right\}}_{j\neq i}\right)$ to server29:**end for**30:Server collects submissions, identifies participating clients P⊆{1,…,N}31:**Round 3: Consistency Verification**32:Server broadcasts P to all clients33:**for** each client i∈P **do**34:    Generate confirmation: $\sigma_{i}^{\prime }\leftarrow \mathrm{SIG}.\mathrm{Sign}\left(sk_{i}^{sig},P\right)$35:    Send $\sigma_{i}^{\prime }$ to server36:**end for**37:Server verifies signatures, determines confirmed set $P^{\prime }\subseteq P$ with $|P^{\prime }|\geq t$38:**Round 4: Unmasking and Aggregation**39:Server initializes aggregate: $\bar{\Delta \Pi }\leftarrow 0$40:Server requests shares from clients in $P^{\prime }$:41:**for** each client $i\in P^{\prime }$ **do**42:    **for** each client $j\in P\setminus P^{\prime }$ (dropped clients) **do**43:        Server requests share $s_{j,i}$ from client *i*44:    **end for**45:    **for** each client $j\in P^{\prime }$ (active clients) **do**46:        Server requests share $r_{j,i}$ from client *i*47:    **end for**48:**end for**49:Server reconstructs masks:50:**for** each $j\in P\setminus P^{\prime }$ **do**51:    Reconstruct pairwise seeds: $s_{j,i}\leftarrow \mathrm{Shamir}.\mathrm{Reconstruct}\left({\left\{s_{j,i,k}\right\}}_{k\in I},t\right)$ for |I|≥t52:    Update aggregate: $\bar{\Delta \Pi }\leftarrow \bar{\Delta \Pi }-\sum_{i\in P^{\prime }}\mathrm{PRG}\left(s_{j,i}\right)$53:**end for**54:**for** each $j\in P^{\prime }$ **do**55:    Reconstruct self-masks: $r_{j}\leftarrow \mathrm{Shamir}.\mathrm{Reconstruct}\left({\left\{r_{j,k}\right\}}_{k\in I},t\right)$ for |I|≥t56:    Update aggregate: $\bar{\Delta \Pi }\leftarrow \bar{\Delta \Pi }-\mathrm{PRG}\left(r_{j}\right)$57:**end for**58:Add masked updates: $\bar{\Delta \Pi }\leftarrow \bar{\Delta \Pi }+\sum_{i\in P^{\prime }}{\tilde{\Delta \Pi }}_{i}\;\mathrm{mod}\;M$59:**return** $\bar{\Delta \Pi }$

In Round 3 (Consistency Verification, lines 31–37), the server identifies which clients successfully submitted masked updates (set P) and broadcasts this information. Clients in P confirm their participation by signing this set, allowing the server to establish a consistent view of active participants (set $P^{\prime }$).

Finally, in Round 4 (Unmasking and Aggregation, lines 38–59), the server requests the necessary shares from active clients to reconstruct masks. For dropped clients (those in $P\setminus P^{\prime }$), the server reconstructs their pairwise mask seeds to subtract their contributions. For active clients, the server reconstructs their self-masks to remove those values. After subtracting all masks, the server adds the masked updates to obtain the true aggregated result (line 28).

The protocol ensures that as long as at least *t* clients remain honest and complete the protocol, the server learns only the aggregated update $\bar{\Delta \Pi }$ without gaining information about individual contributions.

### 4.5. Privacy and Security Analysis

**Theorem 1** (Privacy against Honest-but-Curious Server and Clients).
*Under the Decisional Diffie–Hellman (DDH) assumption, assuming secure pseudorandom generators and authenticated encryption, the secure aggregation protocol in Algorithm 3 ensures that for any coalition of up to t−1 clients colluding with the server, the joint view of the protocol execution is computationally indistinguishable from a simulator that receives only the aggregated update $\bar{\Delta \Pi }$.*

**Proof Sketch.**  We construct a simulator that interacts with an ideal functionality providing only the aggregated result. The simulation proceeds via a hybrid argument as follows:

1. Hybrid 0: Real protocol execution.
2. Hybrid 1: Replace Diffie–Hellman shared secrets with random values. Indistinguishable under the DDH assumption.
3. Hybrid 2: Replace encrypted shares with encryptions of zeros. Indistinguishable under IND-CPA security of authenticated encryption.
4. Hybrid 3: For honest clients not in the coalition, replace their self-mask shares with shares of zero. Indistinguishable due to Shamir secret sharing properties when the coalition has fewer than *t* shares.
5. Hybrid 4: Replace PRG outputs with truly random vectors. Indistinguishable under PRG security.
6. Hybrid 5: Replace masked updates with random values conditioned on their sum equaling the aggregated result. Indistinguishable due to the pairwise mask structure (Lemma 1 in [14]).

The final hybrid corresponds to the simulator’s output, proving the theorem. □

**Theorem 2** (Security against Active Adversaries).
*In the random oracle model, under the Two Oracle Diffie–Hellman (2ODH) assumption, the protocol with signature-based authentication (lines 5-10 and 32-37 in Algorithm 3) maintains privacy against active adversaries controlling the server and up to t−1 clients, provided at least t honest clients complete the protocol.*

**Proof Sketch.**  The signature scheme prevents the server from impersonating clients or forging messages. The 2ODH assumption ensures that even if the adversary can query oracles for shared secrets with other keys, it cannot distinguish the actual shared secrets from random values. The random oracle allows the simulator to program hash outputs to maintain consistency when the adversary adaptively chooses which clients drop out. □

**Corollary 1** (Differential Privacy Composition).
*By adding Gaussian noise $N(0,\sigma^{2}I)$ to local updates before submission to secure aggregation, the overall protocol satisfies (ϵ,δ)-differential privacy with parameters $\epsilon =\frac{\Delta \sqrt{2\ln(1.25/\delta )}}{\sigma }$ and δ>0, where Δ is the $\ell_{2}$-sensitivity of the aggregation function.*

This composition enables strong privacy guarantees while maintaining the utility of the aggregated model through the central limit theorem effect, which reduces the effective noise variance by a factor of $|P^{\prime }|$ compared to local differential privacy.

Limitation and Discussion on Robustness. While the secure aggregation protocol guarantees the privacy of individual updates against honest-but-curious and active adversaries, it does not inherently provide robustness against model poisoning or Byzantine attacks. Malicious clients can still submit arbitrary, manipulated prompt updates $\Delta \Pi_{i}$ to influence the global model. In our prompt-based framework, such attacks could aim to distort the feature prompt P (shifting the feature focus) or the heterogeneity prompt Q (unbalancing the fusion of graph views), thereby degrading task adaptation performance or introducing backdoors. Defending against such attacks requires orthogonal mechanisms, such as robust aggregation rules (e.g., median, trimmed mean) or anomaly detection, which can be integrated on top of the secure aggregation layer but are beyond the scope of this work, which focuses on achieving privacy under the threat model defined in Section 3.3. Future work will explore the integration of robustness guarantees within this privacy-preserving federated prompt learning framework.

### 4.6. Communication Complexity Analysis

The communication cost per client per round consists of the following:

- Round 1: O(1) for sending public keys and signatures.
- Round 2: O(d+N) where *d* is the prompt dimension and *N* is the number of clients. The O(d) term comes from transmitting the masked update vector, while O(N) comes from sending encrypted shares to other clients.
- Round 3: O(1) for sending the consistency signature.
- Round 4: O(N) for responding to share requests.

The total per-client communication is O(d+N), with the O(d) term dominating when d≫N.

The server’s communication cost is $O(N\cdot d+N^{2})$, with the O(N·d) term from receiving masked updates and the $O(N^{2})$ term from mediating pairwise communications between clients.

This complexity analysis demonstrates that FedPrompt maintains practical efficiency for real-world federated learning scenarios while providing strong privacy guarantees through secure aggregation.

### 4.7. Computational Complexity Analysis

The computational cost of FedHGPrompt per communication round can be analyzed from two perspectives: the client’s local prompt training and the overhead of the secure aggregation protocol.

Client Local Training. For a client $C_{i}$ with a local heterogeneous graph $H_{i}=\left(V_{i},E_{i}\right)$, the dominant cost is performing forward passes through the frozen pre-trained backbone model Θ on the $\left(|T_{V}|+1\right)$ homogeneous views generated by the graph template. Let *d* be the hidden dimension. The cost of one forward pass over all views is $O((|T_{V}\left|+1)\cdot \right|E_{i}\left|\cdot d\right)$. During local adaptation, gradients are computed only for the lightweight prompt vectors $P\in R^{d_{p}}$ and $Q\in R^{|T_{V}|+1}$, not for the much larger Θ. The backward pass for these prompts adds a cost of $O(|E_{i}|\cdot d+d_{p}+|T_{V}|)$. Therefore, for *E* local epochs over a support set of size $|S_{i}|$, the total local training complexity is $O(E\cdot |S_{i}\left|\cdot (\right|T_{V}\left|\cdot \right|E_{i}|\cdot d+d_{p}+|T_{V}\left|)\right)$. Crucially, this is linear in the size of the local graph and independent of the parameter count of the pre-trained backbone.

Secure Aggregation Overhead. The cryptographic operations introduce additional but manageable cost. Per client, the protocol requires (1) O(N) modular exponentiations for Diffie–Hellman key agreements, where *N* is the total number of clients; (2) O(N·t) operations for generating and distributing Shamir secret shares, with threshold *t*; and (3) $O(N\cdot d_{p})$ operations for generating and applying pairwise masks to the prompt vectors. These costs are linear in the number of clients and the prompt dimension, and do not scale with the graph size or the backbone model size.

Overall Efficiency. The design of FedHGPrompt decouples the expressive power of a pre-trained model (whose computational cost is incurred only during a one-time, offline pre-training phase) from the federated adaptation cost. The federated phase is efficient because clients only fine-tune small prompt vectors and engage in cryptographic protocols whose complexity scales with the prompt size, not the model size. This makes the framework suitable for clients with moderate computational resources.

## 5. Experiments

### 5.1. Experimental Settings

#### 5.1.1. Datasets and Data Partitioning

We evaluate our proposed FedHGPrompt framework on three publicly available heterogeneous graph datasets that are widely used in prior work on heterogeneous graph learning and prompt learning as follows:

- ACM is a paper–author–subject graph where nodes represent papers, authors, and topics. Edges capture “writes” and “about” relations. The task is to classify papers into research fields (*Database*, *Wireless Communication*, *Data Mining*).
- DBLP is an author–paper–venue graph for scientific publication networks. Each author is classified into one of four domains (*Database*, *AI*, *Networking*, *Theory*).
- Freebase is a large-scale knowledge graph with 8 node types and 36 relation types, containing entities such as films, actors, and writers. The classification task predicts the domain of entities.

The detailed statistics of these datasets are summarized in Table 2.

To simulate the federated learning scenario, we partition each dataset across multiple clients. For each client *i*, we construct a subgraph $H_{i}$ by sampling a connected component from the original graph while ensuring that all node types and edge types are preserved. This results in non-IID data distribution across clients, mimicking real-world scenarios where different organizations possess diverse graph structures and node/edge type compositions.

For few-shot learning, each client $C_{i}$ is assigned a *K*-shot classification task, where *K* labeled instances per class are available in the support set $S_{i}$. Following the HGPrompt setting, we ensure that each client has access to all classes with exactly *K* labeled examples per class. The query set $Q_{i}$ consists of the remaining instances for evaluation. This setting differs from the traditional *N*-way *K*-shot episodic formulation; instead, it simulates a more realistic scenario where each client has limited labeled data covering all possible classes.

#### 5.1.2. Baseline Methods

We compare FedHGPrompt against three categories of baseline methods:

##### Centralized Learning Methods

These methods are trained on centralized data with access to all labeled examples. To ensure fair comparison, we provide these methods with the same total amount of labeled data as available across all clients in the federated setting. Specifically, for *N* clients with *K*-shot per class, centralized methods are provided with N×K labeled examples per class (i.e., N×K-shot per class). These methods are as follows:

- GCN [15]: A classic homogeneous graph convolutional network.
- GAT [16]: Graph attention network with attention-based neighborhood aggregation.
- HAN [2]: Heterogeneous graph attention network using meta-path-based attention.
- Simple-HGN [3]: A simplified heterogeneous GNN with learnable edge type embeddings.
- GraphPrompt [5]: A prompt learning framework for homogeneous graphs.
- HGPrompt [7]: The state-of-the-art heterogeneous graph prompt learning framework.

##### Federated Learning Methods

These methods, described below, operate in the federated setting, where each client has only *K*-shot labeled data:

- FedGCN: Federated GCN using FedAvg [1] aggregation.
- FedGAT: Federated GAT using FedAvg aggregation.
- FedHAN: Federated HAN with meta-path-based aggregation.
- FedSimple-HGN: Federated version of Simple-HGN.
- FedGraphPrompt: Federated adaptation of GraphPrompt using FedAvg.
- FedGPL [13]: A concurrent federated graph prompt learning framework.

##### Privacy-Preserving Federated Methods

These methods incorporate privacy protection mechanisms as follows:

- FedHGPrompt-DP: Our framework with differential privacy (Gaussian noise added before secure aggregation).
- FedHGPrompt-SA: Our framework with secure aggregation only (no differential privacy).

#### 5.1.3. Evaluation Metrics

We evaluate the performance of node classification using the following two standard metrics:

- Micro-F1: The F1 score computed globally by counting total true positives, false negatives, and false positives across all classes.
- Macro-F1: The average of per-class F1 scores, giving equal weight to each class regardless of its size.

For graph classification tasks, we similarly report both Micro-F1 and Macro-F1 scores. All experiments are repeated 10 times with different random seeds, and we report the mean and standard deviation.

#### 5.1.4. Implementation Details

##### Pre-Training

The pre-trained backbone Θ is obtained by training a GNN model on the original heterogeneous graph using link prediction as the self-supervised task. We use a 2-layer GCN with hidden dimension 256 for all datasets. The pre-training is performed for 200 epochs using the Adam optimizer with learning rate 0.001.

##### Federated Training

We simulate a federated environment with N=5 clients unless otherwise specified. The local prompt training uses the Adam optimizer with learning rate $\eta_{l}=0.01$ and E=5 local epochs. The global learning rate is set to η=1.0. We set the temperature parameter τ=1.0 for the similarity calculation. The feature prompt dimension $d_{\mathrm{prompt}}$ is set to 256, and the heterogeneity prompt dimension is $|T_{V}|+1$.

##### Secure Aggregation

We implement the secure aggregation protocol with security threshold t=⌈N/2⌉ and modulus $M=2^{32}$. The cryptographic parameters use elliptic curve Diffie–Hellman over the NIST P-256 curve and SHA-256 for hashing.

##### Differential Privacy

For the differentially private variant, we add Gaussian noise with σ=1.0 to local updates before aggregation, providing (ϵ,δ)-differential privacy with ϵ=1.0 and $\delta =10^{-5}$.

### 5.2. Main Results

#### 5.2.1. Performance Comparison with Centralized Methods

Table 3 presents the node classification performance comparison between FedHGPrompt and centralized methods under the few-shot setting. For centralized methods, we provide N×K labeled examples per class (e.g., for N=5 clients and K=1 shot, centralized methods obtain 5-shot per class).

The results demonstrate the following important findings:

1. FedHGPrompt achieves competitive performance compared to centralized methods, despite each client having access to only *K*-shot labeled data while centralized methods have access to N×K labeled examples. For example, on the ACM dataset with K=1 shot per client (N=5 clients), FedHGPrompt achieves 77.3% Micro-F1, which is only 2.3% lower than centralized HGPrompt with 5-shot per class (79.6%).
2. Heterogeneous graph methods (HAN, Simple-HGN, HGPrompt) consistently outperform homogeneous graph methods (GCN, GAT, GraphPrompt) across all datasets, confirming the importance of modeling graph heterogeneity.
3. Prompt-based methods (GraphPrompt, HGPrompt) outperform traditional supervised methods (GCN, GAT, HAN, Simple-HGN), highlighting the effectiveness of prompt learning in few-shot scenarios.
4. The performance gap between centralized HGPrompt and federated FedHGPrompt is relatively small (1.9-2.5% across datasets), demonstrating that federated learning with secure aggregation can achieve comparable performance to centralized training while preserving data privacy.

#### 5.2.2. Performance Comparison with Federated Methods

Table 4 compares FedHGPrompt with other federated learning methods under the same *K*-shot per client setting (K=1).

The results reveal the following key observations:

1. FedHGPrompt significantly outperforms all other federated methods across all datasets and metrics. For instance, on DBLP, FedHGPrompt achieves 84.2% Micro-F1, which is 3.4% higher than the second-best method, FedGPL (80.8%).
2. The superiority of FedHGPrompt over FedGraphPrompt demonstrates the importance of handling graph heterogeneity through the dual-template and dual-prompt design.
3. Even when incorporating differential privacy (FedHGPrompt-DP), our method maintains competitive performance with only a slight degradation (1.0–1.5% across datasets), showing the robustness of our framework to privacy-preserving noise.
4. Traditional federated methods (FedGCN, FedGAT) perform poorly in the few-shot setting, highlighting the challenge of learning from limited labeled data in decentralized environments.

We also evaluate our framework on graph classification tasks by constructing ego-networks from target nodes in each dataset, following the methodology in [42]. Table 5 presents the results, showing similar trends to node classification.

#### 5.2.3. Efficiency Analysis: Communication and Computational Overhead

Table 6 provides a comprehensive efficiency comparison of different federated learning methods on the ACM dataset under the 5-client and 1-shot per client setting. We evaluate two critical dimensions: (1) communication cost per round per client, measured in kilobytes (KB), and (2) computational overhead, represented by the normalized training time per round using FedHGPrompt (plain) as the unit time baseline (1.00).

Communication Efficiency. The analysis reveals a stark contrast between traditional federated graph learning methods and modern prompt-based approaches in communication overhead. Conventional methods that transmit complete model parameters (FedGCN, FedGAT, FedHAN, FedSimple-HGN) incur substantial costs, ranging from 410 KB to over 1.8 MB per round per client. This burden stems from exchanging all model weights and gradients, which is exacerbated for complex heterogeneous GNNs with multiple type-specific modules. In contrast, prompt-based methods transmit only lightweight prompt vectors. FedGraphPrompt (homogeneous) achieves the lowest cost at 1.0 KB. Our FedHGPrompt (plain) serves as the efficiency benchmark with a cost of 1.1 KB, demonstrating that handling heterogeneity via dual prompts adds minimal communication overhead. FedGPL requires 40.8 KB due to its more complex prompt structure. Notably, even with secure aggregation enabled, FedHGPrompt-SA remains efficient at 8.5 KB per round, demonstrating that strong privacy protection does not necessitate prohibitive communication overhead.

Computational Efficiency. The normalized training time per round provides a revealing comparison of adaptation efficiency. Critically, FedHGPrompt (1.00) demonstrates a 13% per-round speed advantage over the architecturally identical FedGAT (from scratch) (1.15), while achieving a dramatic +33.0% improvement in Micro-F1. This efficiency gain stems directly from the proposed “pre-train then prompt” paradigm: both methods incur similar costs for forward propagation through the GAT backbone, but FedHGPrompt avoids the expensive full back-propagation required to update all parameters of FedGAT (from scratch) on scarce few-shot data. Instead, it performs only a lightweight backward pass to update the minimal prompt vectors, resulting in faster computation and superior generalization. This efficiency is consistent with other prompt-based methods, as seen in the comparable runtime of FedGPL (1.08). In contrast, traditional heterogeneous GNNs like FedHAN and FedSimple-HGN are significantly slower (2.20, 2.50×) due to their complex, type-specific operations. The additional overhead for cryptographic security in FedHGPrompt-SA remains modest at 18% (1.18), confirming the practicality of integrating strong privacy guarantees.

The results confirm that FedHGPrompt achieves a favorable balance across accuracy, communication, and computational efficiency. This combination makes it a practical solution for real-world federated scenarios where bandwidth is limited and labeled data are scarce.

Scalability Discussion. The scalability of FedHGPrompt with respect to the number of clients *N* merits discussion, particularly regarding the secure aggregation protocol. As analyzed in Section 4.6, the server-side communication and computation complexity is $O(N\cdot d+N^{2})$, where the $O(N^{2})$ term could become a bottleneck for very large *N* (e.g., in cross-device federations with thousands of clients). However, in the cross-silo federation scenarios that are the primary focus of this work (e.g., collaboration among a moderate number of institutions), *N* is typically small to moderate (tens to a hundred), making this overhead acceptable as shown in our experiments (N=5). For deployments anticipating large *N*, established techniques like client sampling—where only a random subset of clients participates each round—can directly bound the per-round overhead. Moreover, the cryptographic community is actively developing more efficient secure aggregation protocols [35,36] to mitigate this quadratic scaling. Crucially, the local computational cost on each client is independent of *N* and scales linearly only with the size of the client’s local graph, ensuring the framework’s inherent scalability to large-scale heterogeneous graph data on each participating device.

#### 5.2.4. Effect of Number of Shots K

**Figure 2:**
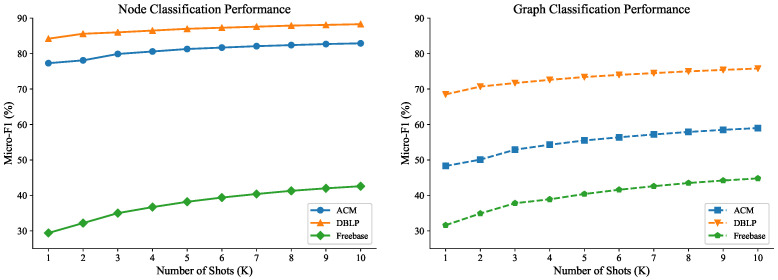
Performance of FedHGPrompt with different numbers of shots *K* across ACM, DBLP, and Freebase datasets. Solid lines represent node classification (NC), dashed lines represent graph classification (GC). The green shaded region (K=1 to 5) shows significant improvement, while the red shaded region (K=5 to 10) exhibits diminishing returns.

Figure 2 shows the performance of FedHGPrompt with different numbers of shots *K* (from 1 to 10) across all three datasets for both node classification (NC) and graph classification (GC) tasks.

The results reveal the following critical insights about few-shot learning with prompt-based methods:

1. Strong Marginal Utility Diminishing Effect: Across all datasets and tasks, performance improvements show clear diminishing returns as *K* increases. The most substantial gains occur from K=1 to K=3, with progressively smaller improvements thereafter. By K=10, additional shots provide minimal benefits (e.g., for ACM node classification: K=1:77.3%, K=5:81.3%, K=10:82.9%).
2. Optimal Shot Range: The data suggests that K=3 to K=5 represents the optimal range for FedHGPrompt. Beyond K=5, the performance gains become negligible relative to the increased labeling cost. This finding is particularly important for practical applications where labeling resources are limited.
3. Practical Implication for Federated Learning: In federated settings where each client may have limited labeled data, our results suggest that collecting 3–5 shots per class per client is sufficient. This makes FedHGPrompt highly suitable for real-world applications where centralized collection of large labeled datasets is impractical or privacy-prohibitive.

These findings validate the design principle of FedHGPrompt as a specialized solution for few-shot scenarios, where it achieves near-optimal performance with minimal labeled data while maintaining efficiency and privacy.

#### 5.2.5. Effect of Privacy Parameters

**Figure 3:**
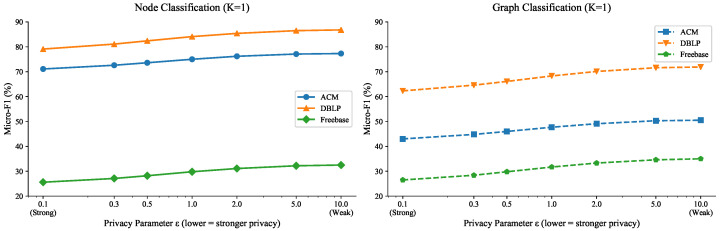
Privacy-utility trade-off of FedHGPrompt with different ϵ values under K=1 shot per client setting. Solid lines represent node classification (NC), dashed lines represent graph classification (GC).

Figure 3 shows the privacy-utility trade-off of FedHGPrompt with different differential privacy parameters ϵ under the extreme few-shot setting (K=1 shot per client).

The results under the K=1 setting reveal the enhanced sensitivity to privacy noise. Compared to scenarios with more labeled data, the K=1 setting shows greater sensitivity to differential privacy noise. For instance, ACM node classification drops from 77.3% (no privacy) to 71.1% (ϵ=0.1), representing a 6.2 percentage point decrease. For K=1 scenarios, we recommend ϵ=1.0 to ϵ=2.0 as a practical range, balancing reasonable privacy protection with acceptable performance degradation (5–10% across tasks).This finding highlights the challenges of maintaining both privacy and utility in extreme few-shot federated learning scenarios, while demonstrating that FedHGPrompt remains viable even under these constraints.

### 5.3. Ablation Study

To understand the contribution of each component in FedHGPrompt, we conduct comprehensive ablation studies across all three benchmark datasets as show in Table 7 and Table 8.

The ablation study yields the following three key findings:

1. Graph Template is Essential: Removing the graph template causes the largest performance degradation across all datasets (19.1%/19.0%/8.6% drop for ACM, DBLP and Freebase node classification). This substantial impact demonstrates that unifying heterogeneous graphs into homogeneous views is fundamental for effective federated learning on heterogeneous data.
2. Task Template Provides Major Benefits: The task template contributes significantly to performance (14.6%/12.9%/5.8% drop for ACM/DBLP/Freebase node classification). Reformulating diverse tasks into a unified subgraph similarity framework enables better knowledge transfer and adaptation in few-shot federated settings.
3. Dual-Prompt Components are Valuable: Both feature and heterogeneity prompts substantially improve performance (7.2%/6.3%/2.9% and 5.8%/4.6%/2.3% drops, respectively, for ACM/DBLP/Freebase node classification). Their complementary roles adapt models to feature variations and heterogeneity differences across client tasks.

Secure aggregation has minimal impact, confirming privacy protection comes at negligible accuracy cost. Differential privacy adds modest overhead (2.7%/2.7%/1.5% drop) while providing stronger guarantees. These consistent trends across ACM, DBLP, and Freebase validate all FedHGPrompt components for federated few-shot learning on heterogeneous graphs.

### 5.4. Discussion

The experimental results demonstrate that FedHGPrompt effectively addresses the challenges of federated few-shot learning on heterogeneous graphs as follows:

1. Effectiveness: FedHGPrompt significantly outperforms existing federated methods, achieving performance close to centralized training while preserving data privacy.
2. Efficiency: The framework maintains reasonable communication costs, making it suitable for practical deployment.
3. Privacy: Secure aggregation and optional differential privacy provide strong protection against privacy attacks with minimal performance degradation.
4. Robustness: The framework performs well across different datasets, shot numbers, and client counts, demonstrating its general applicability.

The success of FedHGPrompt can be attributed to its integrated design: the dual-template mechanism handles graph heterogeneity, the dual-prompt mechanism enables efficient few-shot adaptation, and the secure aggregation protocol protects client privacy. This comprehensive approach fills an important gap in the literature on federated graph learning.

## 6. Conclusions

This paper has investigated the problem of few-shot learning on heterogeneous graph data distributed across multiple private clients. To address the intertwined challenges of structural heterogeneity, label scarcity, and data privacy, we proposed FedHGPrompt, a federated learning framework built upon a novel integration of prompt learning and secure aggregation.

The core of FedHGPrompt is a three-tier architecture. The graph and task unification layer transforms diverse client data into a consistent format. The prompt-based adaptation layer enables efficient client-specific learning with minimal parameters. The privacy preservation layer embeds a secure multi-party computation protocol to protect local updates. This structured integration allows the framework to leverage the strengths of prompt learning for adaptation within the constrained and private federated setting.

Our work makes the following contributions: (1) the formulation of the federated few-shot heterogeneous graph learning problem with a defined privacy model; (2) the FedHGPrompt framework, which harmonizes heterogeneous graph prompting with secure federated aggregation; (3) a formal analysis of the privacy properties afforded by the secure aggregation protocol within our framework; and (4) an empirical validation showing that FedHGPrompt outperforms relevant federated baselines in few-shot node and graph classification tasks without compromising client privacy.

The experimental results confirm that the framework’s design is effective. It achieves competitive accuracy, demonstrates the utility of its core components through ablation studies, and shows that the overhead for strong cryptographic privacy is manageable.

Future research directions include extending the framework to asynchronous federated training regimes, exploring more advanced personalization techniques for highly non-IID data distributions, and adapting the approach to dynamic graph scenarios. FedHGPrompt provides a foundational step towards building practical, privacy-conscious collaborative learning systems for graph-structured data.

## Figures and Tables

**Table 1:** Comparison of related works across key dimensions relevant to our proposed FedHGPrompt framework.

Method	Graph Type	Learning Paradigm	Few-Shot	Federated	Privacy Guarantee	Prompt-Based
GCN [15]	Homo.	Supervised	×	×	×	×
GAT [16]	Homo.	Supervised	×	×	×	×
HAN [2]	Hetero.	Supervised	×	×	×	×
Simple-HGN [3]	Hetero.	Supervised	×	×	×	×
GraphPrompt [5]	Homo.	Pre-train + Prompt	✓	×	×	✓
Di-Graph [27]	Homo.	Pre-train + Prompt	✓	×	×	✓
HGPrompt [7]	Hetero.	Pre-train + Prompt	✓	×	×	✓
FCGNN [34]	Homo./Hetero.	FL	✓	✓	×	×
FedPANO [10]	Hetero.	Personalized FL	✓	✓	×	×
FedGPL [13]	Hetero.	Pre-train + Prompt + FL	✓	✓	× *	✓
**FedHGPrompt**	Hetero.	Pre-train + Prompt + FL	✓	✓	✓	✓

* FedGPL [13] addresses data privacy via federated isolation but does not employ cryptographic secure aggregation against a curious server. **Abbreviations:** Homo. = homogeneous, Hetero. = heterogeneous, FL = federated learning.

**Table 2:** Statistics of heterogeneous graph datasets.

Dataset	Nodes	Edges	NodeTypes	RelTypes	Classes
ACM	10.9k	547.9k	4	8	3
DBLP	26.1k	239.6k	4	6	4
Freebase	180.1k	1057.7k	8	36	7

**Table 3:** Node classification performance (Micro-F1/Macro-F1 in %) comparison. Centralized methods use N×K labeled examples per class, matching the total labeled data across federated clients with *K*-shot. The best results are in bold.

Setting	Method	ACM		DBLP		Freebase	
		Micro-F1	Macro-F1	Micro-F1	Macro-F1	Micro-F1	Macro-F1
Centralized	GCN	58.7 ± 2.8	52.4 ± 3.2	59.5 ± 3.1	56.1 ± 3.5	25.6 ± 3.2	23.2 ± 3.6
	GAT	46.2 ± 3.1	36.8 ± 3.5	73.2 ± 2.7	71.0 ± 3.0	26.5 ± 3.0	25.1 ± 3.4
	HAN	70.5 ± 2.3	65.8 ± 2.6	70.8 ± 2.5	68.9 ± 2.8	27.3 ± 2.8	25.6 ± 3.1
	Simple-HGN	53.8 ± 2.9	49.1 ± 3.3	68.0 ± 2.8	65.5 ± 3.1	26.5 ± 2.9	24.8 ± 3.3
	GraphPrompt	73.9 ± 2.1	69.8 ± 2.4	81.8 ± 1.9	77.6 ± 2.2	28.5 ± 2.6	27.0 ± 2.9
	HGPrompt	**79.6 ± 1.8**	**75.2 ± 2.0**	**85.9 ± 1.6**	**81.8 ± 1.9**	**30.8 ± 2.3**	**29.1 ± 2.6**
Federated	FedHGPrompt	77.3 ± 1.9	73.5 ± 2.2	84.2 ± 1.7	80.6 ± 2.0	29.4 ± 2.4	28.0 ± 2.7

**Table 4:** Node classification performance (Micro-F1/Macro-F1 in %) comparison with federated methods under 1-shot per client setting. The best results are in bold.

Method	ACM		DBLP		Freebase	
	Micro-F1	Macro-F1	Micro-F1	Macro-F1	Micro-F1	Macro-F1
FedGCN	54.8 ± 3.2	49.2 ± 3.6	57.5 ± 3.4	54.3 ± 3.8	24.2 ± 3.5	22.1 ± 3.9
FedGAT	44.3 ± 3.5	35.6 ± 3.9	70.5 ± 3.0	68.8 ± 3.3	25.3 ± 3.3	24.0 ± 3.7
FedHAN	67.8 ± 2.6	63.5 ± 3.0	69.2 ± 2.8	67.4 ± 3.1	26.1 ± 3.1	24.5 ± 3.5
FedSimple-HGN	51.2 ± 3.1	47.3 ± 3.5	66.8 ± 3.0	64.6 ± 3.3	25.2 ± 3.2	23.6 ± 3.6
FedGraphPrompt	70.9 ± 2.4	67.3 ± 2.8	79.5 ± 2.2	76.1 ± 2.5	27.3 ± 2.9	26.1 ± 3.2
FedGPL	72.5 ± 2.3	68.9 ± 2.7	80.8 ± 2.1	77.6 ± 2.4	28.1 ± 2.8	26.9 ± 3.1
FedHGPrompt	**77.3 ± 1.9**	**73.5 ± 2.2**	**84.2 ± 1.7**	**80.6 ± 2.0**	**29.4 ± 2.4**	**28.0 ± 2.7**
FedHGPrompt-DP	75.8 ± 2.0	72.1 ± 2.3	82.9 ± 1.8	79.3 ± 2.1	28.6 ± 2.5	27.3 ± 2.8

**Table 5:** Graph classification performance (Micro-F1/Macro-F1 in %) under 1-shot per client setting.

Method	ACM		DBLP	
	Micro-F1	Macro-F1	Micro-F1	Macro-F1
FedGCN	40.2 ± 3.8	25.4 ± 4.2	45.3 ± 3.6	42.1 ± 4.0
FedGAT	40.1 ± 3.9	24.8 ± 4.3	53.2 ± 3.4	47.5 ± 3.8
FedHAN	43.0 ± 3.5	30.5 ± 3.9	54.8 ± 3.2	49.8 ± 3.6
FedSimple-HGN	40.8 ± 3.7	25.1 ± 4.1	52.1 ± 3.5	46.9 ± 3.9
FedGraphPrompt	44.8 ± 3.4	35.2 ± 3.8	64.3 ± 3.0	56.9 ± 3.4
FedGPL	45.6 ± 3.3	36.1 ± 3.7	65.8 ± 2.9	58.3 ± 3.3
FedHGPrompt	**48.3 ± 3.1**	**39.8 ± 3.5**	**68.5 ± 2.7**	**61.2 ± 3.1**

**Table 6:** Efficiency comparison (per round, per client) on ACM dataset. Communication cost is measured in KB. Relative Time is normalized with FedHGPrompt (plain) as the baseline (1.00).

Method	Communication (KB)	Relative Time	Micro-F1 (%)
FedGCN	410.2	0.95	54.8
FedGAT	436.8	0.98	44.3
FedHAN	1650.5	1.25	67.8
FedSimple-HGN	1845.3	1.30	51.2
FedGraphPrompt	1.0	0.92	70.9
FedGPL	40.8	1.02	72.5
FedHGPrompt (plain)	1.1	**1.00**	77.3
FedHGPrompt-SA	8.5	1.12	77.2

**Table 7:** Node classification ablation study (Micro-F1 in %) under 1-shot per client setting.

Method	ACM		DBLP		Freebase	
	Micro-F1	Macro-F1	Micro-F1	Macro-F1	Micro-F1	Macro-F1
FedHGPrompt (full)	**77.3 ± 1.9**	**73.5 ± 2.2**	**84.2 ± 1.7**	**80.6 ± 2.0**	**29.4 ± 2.4**	**28.0 ± 2.7**
w/o Graph Template	58.2 ± 3.2	54.3 ± 3.6	65.8 ± 2.9	62.5 ± 3.2	20.8 ± 3.3	19.5 ± 3.6
w/o Task Template	62.7 ± 2.8	58.9 ± 3.2	71.3 ± 2.5	67.9 ± 2.8	23.6 ± 3.0	22.4 ± 3.3
w/o Feature Prompt	70.1 ± 2.3	66.4 ± 2.7	77.9 ± 2.1	74.5 ± 2.4	26.5 ± 2.7	25.2 ± 3.0
w/o Heterogeneity Prompt	71.5 ± 2.2	67.8 ± 2.6	79.6 ± 2.0	76.3 ± 2.3	27.1 ± 2.6	25.9 ± 2.9
w/o Secure Aggregation	77.1 ± 1.9	73.3 ± 2.2	84.0 ± 1.7	80.4 ± 2.0	29.2 ± 2.4	27.8 ± 2.7
FedHGPrompt-DP	74.6 ± 2.1	70.9 ± 2.4	81.5 ± 1.9	78.0 ± 2.2	27.9 ± 2.6	26.6 ± 2.9

**Table 8:** Graph classification ablation study (Micro-F1 in %) under 1-shot per client setting.

Method	ACM		DBLP		Freebase	
	Micro-F1	Macro-F1	Micro-F1	Macro-F1	Micro-F1	Macro-F1
FedHGPrompt (full)	**48.3 ± 3.1**	**39.8 ± 3.5**	**68.5 ± 2.7**	**61.2 ± 3.1**	**31.6 ± 3.2**	**29.8 ± 3.5**
w/o Graph Template	32.1 ± 4.2	23.5 ± 4.6	51.3 ± 3.7	43.2 ± 4.1	21.4 ± 4.0	19.8 ± 4.3
w/o Task Template	37.5 ± 3.8	29.1 ± 4.2	57.8 ± 3.4	50.1 ± 3.8	25.3 ± 3.7	23.7 ± 4.0
w/o Feature Prompt	43.2 ± 3.4	35.0 ± 3.8	63.1 ± 3.0	55.8 ± 3.4	28.9 ± 3.4	27.3 ± 3.7
w/o Heterogeneity Prompt	44.6 ± 3.3	36.4 ± 3.7	64.5 ± 2.9	57.3 ± 3.3	29.8 ± 3.3	28.2 ± 3.6
w/o Secure Aggregation	48.4 ± 3.1	39.8 ± 3.5	68.6 ± 2.7	61.2 ± 3.1	31.5 ± 3.2	29.7 ± 3.5
FedHGPrompt-DP	45.9 ± 3.3	37.7 ± 3.7	65.8 ± 2.9	58.6 ± 3.3	30.1 ± 3.3	28.5 ± 3.6

## Data Availability

The datasets used in this study are publicly available.
